# An Embodied Cognition Perspective on the Role of Interoception in the Development of the Minimal Self

**DOI:** 10.3389/fpsyg.2021.716950

**Published:** 2021-10-12

**Authors:** Lisa Musculus, Markus R. Tünte, Markus Raab, Ezgi Kayhan

**Affiliations:** ^1^Institute of Psychology, German Sport University Cologne, Cologne, Germany; ^2^Department of Developmental and Educational Psychology, Faculty of Psychology, University of Vienna, Vienna, Austria; ^3^School of Applied Sciences, London South Bank University, London, United Kingdom; ^4^Department of Developmental Psychology, University of Potsdam, Potsdam, Germany; ^5^Max Planck Institute for Human Cognitive and Brain Sciences, Leipzig, Germany

**Keywords:** interoception, bodily self, embodied cognition, cardioception, development of minimal self

## Abstract

Interoception is an often neglected but crucial aspect of the human minimal self. In this perspective, we extend the embodiment account of interoceptive inference to explain the development of the minimal self in humans. To do so, we first provide a comparative overview of the central accounts addressing the link between interoception and the minimal self. Grounding our arguments on the embodiment framework, we propose a bidirectional relationship between motor and interoceptive states, which jointly contribute to the development of the minimal self. We present empirical findings on interoception in development and discuss the role of interoception in the development of the minimal self. Moreover, we make theoretical predictions that can be tested in future experiments. Our goal is to provide a comprehensive view on the mechanisms underlying the minimal self by explaining the role of interoception in the development of the minimal self.

## Interoception and the Bodily Minimal Self

Body representation in humans is subsumed under the so-called bodily or minimal self, which is defined as a “person’s phenomenal experience in the here and now” (Hafner et al., 2017, p. 1; Gallagher, 2000). The bodily or minimal self of humans is heavily dependent on the “embedded body” (Gallagher, 2000, p. 15). The minimal self consists of the sense of ownership, which refers to the feeling that one’s body belongs to oneself, and the sense of agency, which is the feeling that one’s actions cause effects (Gallagher, 2000; Verschoor and Hommel, 2017). Given the crucial role of the body in conceptualizing the sense of ownership and the sense of agency, and hence the human minimal self, it is surprising that *internal* bodily signals such as heartbeat and respiration have been largely ignored in this line of research (Tsakiris et al., 2011; Marshall et al., 2018; Seth and Tsakiris, 2018). For instance, a newborn’s heart beats at ca. 127 beats per minute (bpm) increasing to a maximum of ca. 145bpm within 1month, before it decreases to 112bpm by the age of 2 years (Fleming et al., 2011). Heartbeat perception is central to research on interoception, which is traditionally defined as the perception and sensation of the internal bodily signals (Murphy et al., 2017). From an embodied cognition perspective, it seems implausible that such bodily changes during development would not affect the body representation, hence the minimal self. In this paper, we argue that interoceptive signals are fundamental to the phenomenal experience of here and now constructing the minimal self. Grounding our arguments on the embodiment framework, we discuss how interoception shapes the development of the minimal self in humans.

Our perspective aims to extend the embodied cognition account of interoceptive inference (Marshall et al., 2018) by explicitly focusing on the role of interoception in the *development* of the human minimal self. The call for the research topic postulates embodied cognition as a powerful framework in explaining the minimal self (Hafner et al., 2021). Embodied cognition accounts are manifold (for detailed overviews see, e.g., Wilson, 2002; Shapiro, 2019) varying regarding central assumption and their “radicalism” (Raab and Araujo, 2019, p. 1) with respect to whether the link between the environment and perception, cognition and action is direct (e.g., Gibson, 1979; Chemero, 2011; Jacob, 2016) or mediated through representations (e.g., Newen et al., 2018). We base our perspective on the central assumption that representations benefit human’s flexible and adaptive way of acting in a complex world (Schulz, 2018). We thereby take a “moderate” position (cf., Goldman, 2012), acknowledging at the same time that other approaches exist aiming to overcome the separation of approaches (e.g., Witt and Riley, 2014; Ciaunica et al., 2021). In addition, our embodied cognition perspective considers bodily changes relevant to explaining human development (Musculus et al., 2021), and, here, relate it to the development of the self.

Our contribution consists of a comparative overview of the central theoretical accounts explaining the link between interoception and the bodily minimal self (Marshall et al., 2018; Seth and Tsakiris, 2018). Based on this comparison, we present our embodied cognition perspective in more detail focusing on the emerging minimal self. Following a discussion of empirical findings on how interoception shapes the development of the bodily minimal self, we will outline theoretical predictions and a research program to better understand the role of interoception in the development of the bodily minimal self from our embodied cognition perspective.

### Comparative Overview of Theoretical Accounts

In this part, we will compare two theoretical accounts that explain the role of interoception in the bodily minimal self on three levels (i.e., origin, central model assumptions, relation of interoception to the self).

The instrumental interoceptive inference account, proposed by Seth and Tsakiris (2018), originates from cybernetics and the free-energy principle. According to the instrumental interoceptive inference account (motor) actions serve the regulation of interoceptive states through a hierarchically organized generative model (Seth et al., 2012; Seth and Tsakiris, 2018): The generative model encodes priors of sensory information in higher levels of the neural hierarchy, based on which lower-level information such as interoceptive states are predicted. These top-down predictions are compared to the perceived interoceptive states. The difference between the predicted and the perceived states results in prediction errors, which are then sent back to the higher levels in the hierarchy to further update the generative models (Seth et al., 2012; Seth and Tsakiris, 2018). Through repetition of this hierarchical cascading, interoceptive prediction errors are minimized, which eventually maximizes the interoceptive generative models. These models form the basis of a sense of self and the experience of selfhood (Seth et al., 2012). Importantly, interoceptive prediction errors can also be minimized through action, also known as active inference. In the case of interoception, this refers to “intero-actions” (e.g., reflexes). Together, interoceptive (active) inference serves the overall goal of allostasis: maintaining physiological parameters of the body within a constant range by adapting to environmental change (Sterling, 2014; Seth and Tsakiris, 2018). This notion draws the connection to the experience of selfhood: Interoception fosters the stability of the bodily minimal self as opposed to the ever-changing exteroceptive information (Tsakiris, 2017).

Marshall et al. (2018) built up on this account and elaborated further on the functional link between interoception and (motor) actions. This approach is strongly influenced by cognitive psychology and cognitive neuroscience. According to Marshall et al. (2018), both motor and interoceptive states can form predictions about each other. Predictions are then compared to afferent, sensory input stemming from the sensorimotor system in the case of the motor prediction, and the autonomic system in the case of interoceptive predictions. Importantly, motor and interoceptive predictions are weighed equally in how they contribute to subjective experience emphasizing a functional bidirectional link. This also draws the connection to the experience of selfhood: Interoceptive states modulate the experience of selfhood just as strongly as (motor) actions (Marshall et al., 2018).

### Embodiment Suggests a Bidirectional Link

Both theoretical accounts, although originating from different domains, share the idea that predictive coding can be considered as the “mechanistic process […] forming an initial, theoretical link between” (Marshall et al., 2018, p. 2) interoception and the minimal self. The accounts differ in how they elaborate on the functional relationship between interoception and motor processes. From an instrumental interoceptive inference account, the impact of motor predictions on interoceptive states has been formulated in terms of a hierarchically organized generative model (Seth et al., 2012). This was extended theoretically by explicitly suggesting a bidirectional link in which interoceptive states also predict motor actions (Marshall et al., 2018). We find the theoretical argument of bidirectionality plausible in line with the general tenets of the embodied cognition perspective.

**Figure 1:**
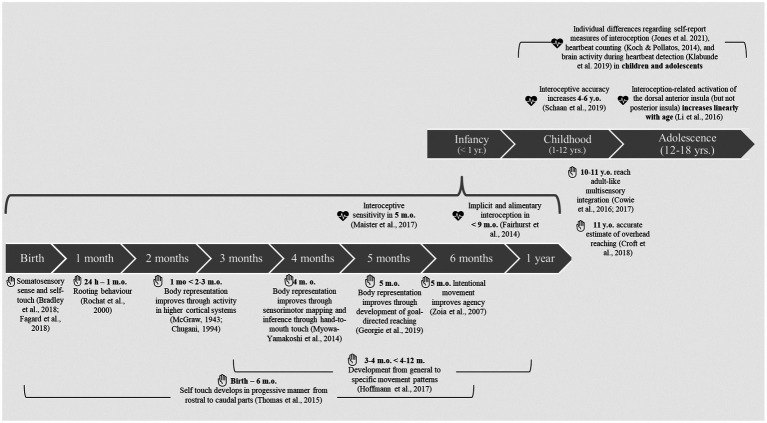
Overview of studies on the development of interoception as well as body representation, multisensory integration, ownership, and agency relevant for the human minimal self during infancy, childhood, and adolescence. The hand symbol represents studies on body representation, multisensory integration, ownership, and agency. The heart symbol represents studies on interoception. The lower part of the figure summarizes the results of infant studies and the upper part of the figure summarizes the study results on children and adolescents. m.o.,month-olds; y.o.,year-olds.

Although both theoretical accounts mention and acknowledge the relevance of a developmental approach, neither of them focus on development in more detail. We tap into this gap and discuss the *development of the minimal self*. Recent reviews on this topic studied the development of the minimal self through experiencing and interacting with the external world (Georgie et al., 2019; Nguyen et al., 2021). We extend this line of research by considering the role of interoception in the development of the minimal self. In particular, we derive theoretical predictions on the developmental trajectory of interoception and discuss its relation to minimal-self dimensions such as the sense of ownership and agency from our embodied cognition perspective. To do so, we summarize the evidence on the development of body ownership and agency in Figure 1 (based on Georgie et al., 2019) and integrate these findings with the development of interoception.

## Development of Interoception and the Minimal Self

Interoception refers to perceiving signals from inner organs such as heartbeat, hunger, or breathing (Herbert and Pollatos, 2012). Interoception also includes the monitoring of these internal states during ongoing activities aiming at keeping the bodily system stable (Craig, 2008; Herbert and Pollatos, 2012; Tsakiris, 2017; Seth and Tsakiris, 2018). Before discussing the development of interoception, we would like to note that we differentiate interoceptive sensitivity from interoceptive awareness. Whereas interoceptive sensitivity can be defined as the implicit detection and discrimination of interoceptive signals, interoceptive awareness is a meta-cognitive process reflecting the explicit evaluation of interoceptive states (Murphy et al., 2017). We consider the findings on the development of interoception from this point of view.

### Empirical Findings on the Development of Interoception

Similar to research on interoception and its role in the minimal self in adulthood (Herbert and Pollatas, 2012; Tsakiris, 2017; Marshall et al., 2018), research on interoception development has mainly focused on heartbeat perception. In this section, we will first present the developmental changes in the frequency of heartbeats, which will be followed by a review on cardiac interoception in infants, children, and adolescents. An overview of this review can be found in Figure 1.

Developmental changes in heartbeat frequency can be divided into four phases: (1) from birth to 1month of age during which the heart rate increases; (2) from 1month to 2 years of age, in which the heart rate decreases steeply; (3) from 2 to 6 years, in which the heart rate decreases but less strongly as compared to (4) 6–12 years of age (Fleming et al., 2011). Thus, from birth to childhood up until 12 years of age, pronounced changes occur in the frequency of heartbeats. Similar developmental changes have been documented for cardiac interoceptive abilities (Koch and Pollatos, 2014; Georgiou et al., 2015; Klabunde et al., 2019; Jones et al., 2021).

In infancy (up to 1year) and early childhood (1–5 years), very few empirical studies investigated interoceptive abilities (Fairhurst et al., 2014; Maister et al., 2017). The only published empirical study investigating cardiac interoception in infants suggests that, already by 5months of age, infants show sensitivity to their cardiac signals (Maister et al., 2017). In this study, infants were presented with images that moved synchronously or asynchronously with their own heartbeat. Infants looked significantly longer at asynchronously presented stimuli suggesting that they were able to distinguish asynchronous from synchronous stimuli (Maister et al., 2017). Moreover, individual differences in looking times were correlated with heartbeat-evoked potentials, a brain signal related to cardiac interoceptive processing (Coll et al., 2021). In other words, infants who responded to the synchronous manipulation also showed stronger neural responses captured by the heartbeat-evoked potentials. These findings support the argument that interoception may contribute to the development of the minimal self.

In children (>5 to 12 years) and adolescents (12–18 years), interoception has been investigated mostly by adopting approaches and methodologies used in adult populations. Empirical findings suggest that, similar to adults, children and adolescents show individual differences in heartbeat counting tasks (Koch and Pollatos, 2014) and self-report measures of interoception such as those collected through the Multidimensional Assessment of Interoceptive Awareness Questionnaire (Jones et al., 2021). By inducing cardiac perturbation through jumping jacks and assessing heartbeat counting abilities before and after the tasks, researchers have shown that children accurately count their heartbeats as early as 4–6 years of age (Schaan et al., 2019). Moreover, brain areas such as the left insula, cuneus, inferior parietal lobule, and prefrontal regions are activated during a heartbeat detection task already at 6 years of age (Klabunde et al., 2019).

Studies in children and adolescents also indicated age-related differences in interoception. For example, children’s performance in an adapted version of the heartbeat counting task increases with age, which marginally predicts emotion regulation, but not emotion recognition (Koch and Pollatos, 2014). Moreover, during the heartbeat detection task adolescents show increased activation in brain regions related to meta-cognition such as the dorsal anterior cingulate cortex, orbital frontal cortex, and mid-inferior frontal gyrus as compared to children (Klabunde et al., 2019). This neural pattern of activation might suggest that meta-cognitive aspects of interoceptive processing might develop throughout adolescence.

Overall, the empirical results describing the developmental trajectory of interoception in childhood, and especially in infancy, are scarce but much needed. Among others, this scarcity of research is likely due to methodological challenges in measuring interoception in younger children. Next, we extend the existing embodiment account on interoception and formulate theoretical predictions on the development of interoception for future research.

### Theoretical Predictions

In the following, we formulate developmental predictions derived from an embodied cognition account of interoceptive inference. Importantly, our embodied cognition perspective assumes that representations form the body-goal link (cf., Pacherie, 2018; Raab and Araujo, 2019; see Witt and Riley, 2014 for alternative accounts considering interoception), enable goal-directed acting in a flexible and adaptive manner (Schulz, 2018), as well as emerge through sensorimotor and bodily experiences throughout development (cf., Musculus et al., 2021). Given the scarcity of research on the development of interoception, and particularly on interoceptive modalities such as respiration, thermoregulation and so forth, we center our arguments on cardiac interoception from birth to 12 years of age. We focus on this age range based on (1) the developmental changes in the frequency of heartbeats (Fleming et al., 2011), (2) motor and bodily development (Musculus et al., 2021), and (3) findings on multisensory integration, the sense of ownership and agency (see Figure 1; cf., Georgie et al., 2019). We point out the interaction between multisensory integration of external sensory input, ownership, and agency with internal bodily signals in the formation of the minimal self in development.

Interoceptive sensitivity is observed in the first months of life (Maister et al., 2017). Interestingly, changes in interoceptive sensitivity coincide with the improvements in sensorimotor mapping such as hand-to-mouth touch (Myowa-Yamakoshi and Takeshita, 2006) and goal-directed reaching (Georgie et al., 2019). Together, these developments might contribute to the formation of body representation, and hence, to the sense of ownership in infants at 5–6months of age (see Figure 1). Through improvements in motor skills and continuous exploration, infants learn to act in a goal-directed manner (i.e., goal-directed touching and reaching). This, in turn, helps them to learn about their body boundaries and relate body-directed goals (e.g., reaching the mouth) to goals in the environment. Establishing this relation might pave the way to a sense of body ownership in humans.

Moving further in the developmental trajectory, we hypothesize that the first 2 years of life are crucial to study the development of interoception. This prediction is based on the rapid decrease in heart-beat frequency until 2 years of age (Fleming et al., 2011) and the rather general developmental embodied cognition premise that phases of rapid bodily changes and motor development promote perceptual and cognitive changes (Loeffler et al., 2016; Musculus et al., 2021). We further hypothesize that there might be more drastic changes in interoceptive sensitivity between 2 and 6 years of age (i.e., phases of rapid growth and motor learning) as compared to 6–12 years of age. Moreover, we expect interoceptive awareness to develop during late childhood to adolescence. This change is likely due to the development of meta-cognitive processes (Klabunde et al., 2019). The developmental changes in interoception coincide with improvements in multisensory integration (Cowie et al., 2016, 2017) and accuracy of reach estimations (Croft et al., 2018), which might indicate more accurate representation of the body–environment relation. This relation might be further mediated by an increase in confidence in judging bodily as well as motor competences.

Further, we specify the relationship between interoception and other minimal-self dimensions such as the sense of ownership and agency. To do so, we dissociate a low-level agency (i.e., agency feeling) from a high-level agency (i.e., agency judgment; Synofzik et al., 2008). We assume that this distinction develops with age. First, we hypothesize that interoceptive sensitivity and body ownership are functionally and reciprocally interconnected. That is, improvements in perceiving and identifying internal bodily signals (i.e., interoception) as well as the boundary between one’s body and the external environment (i.e., body ownership) should benefit one another. For example, perceiving one’s heartbeat might promote the feeling of the body as one’s own. Moreover, we hypothesize that improvements in interoceptive awareness in late childhood or adolescence could coincide with a high-level agency judgment due to the involvement of meta-cognitive processes. Overall, we argue that considering the interaction between interoception, other minimal-self components and bodily development is crucial to define, test, and disentangle mechanisms underlying minimal-self development.

### Future Research and Conclusion

We suggest a research program to empirically test the predictions on the development of interoception. The program entails specific study designs and a psychophysiological multi-method approach to capture the developmental trajectory of interoception as well as its relation to other minimal-self components such as ownership and agency.

We need longitudinal designs to test the developmental trajectories. Longitudinal designs enable us to disentangle intraindividual changes over the course of development as well as interindividual differences when people of the same age develop differently. Moreover, training studies would inform our understanding of the relationship between bodily changes and interoception. In training studies different training groups differentially targeting the bodily system could be implemented to look at the respective effects on interoception. For instance, infants and children could engage in physical exercises that either lead to an increase or a decrease in their heart rate and the respective effects on interoceptive abilities could be measured.

To investigate the link between interoception and other minimal-self components such as ownership and agency, measurements from both lines of research need to be combined. Therefore, we suggest that interoception paradigms should be jointly implemented with body representation (cf., Suzuki et al., 2013) and multisensory integration paradigms in infant and child studies (e.g., Cowie et al., 2016, 2017). Studies combining measures within the same developmental study would improve our understanding of how multiple sources of bodily and sensory information contribute to the development of the self. This would allow us to better understand how ownership and agency relate to and change in relation to interoception.

In combination, developmental study designs and a psychophysiological multi-method approach (Hoffmann et al., 2018) could even help testing potentially competing mechanisms (Marshall et al., 2018; Seth and Tsakiris, 2018) on the relation between interoception and (motor) action and their respective contribution to the minimal self. Combining cohort-longitudinal designs by enrolling infants and children of different ages with simultaneously applying cardiac-physiological (electrocardiography), neural (electroencephalography), and motor (electromyography) measures might help disentangle these mechanisms. In particular, event-related, reaction-time paradigms could be used that require a motor response. At the same time cardiac and motor measures could be combined to infer how interoceptive and motor states functionally interact in the same experimental task.

There are other developmental aspects that we do not elaborate on due to our focus on childhood rather than infancy. However, we deem the following aspects relevant for future work on interoception: The relation between interoception and active self-touch as well as the role of social interactions. Infancy work has lately also considered the link between interoception and haptic perception (i.e., active self-touch; Fotopoulou and Tsakiris, 2017). This work suggests that active self-touch might benefit the later integration of tactile-proprioceptive and visual information relevant for minimal-self development (see Nguyen et al., 2021 for a review). Besides, social interactions have been considered to play a crucial role in the development of the minimal self, particularly in the development of interoceptive abilities in early infancy (Fotopoulou and Tsakiris, 2017). Given that infants are born with limited motor skills, they depend on others to regulate their own bodily needs such as hunger. Thus, infants rely on embodied interactions with their caregivers in order to regulate their interoceptive states. These interactions allow them to learn the regularities within and outside their bodies (Tsakiris, 2017). Future studies should empirically test the role of embodied interactions in the construction of the minimal self early on in life, including all aspects such as interoception, agency, and ownership.

To sum up, a comprehensive research program is warranted. Such a program would further benefit from a new psychophysiological approach (Hoffmann et al., 2018) and from studying social aspects of interoception (Fotopoulou and Tsakiris, 2017). Together, we hope that the theoretical predictions and the research program introduced in this perspective will promote future research to understand the role of interoception in the development of the minimal self.

## Author Contributions

LM, MT, MR, and EK contributed to the conceptualization and wrote and edited the manuscript. All authors contributed to the article and approved the submitted version.

## Funding

LM and MR were funded by the German Research Foundation (DFG; RA 940/21-1). EK was funded by the DFG (grant number: 402789467) and the Open Access Publishing Fund of University of Potsdam. The funders had no role in conceptualization, and development or writing of the manuscript. MT was funded by the Austrian Science Fund (FWF), project number 33486-B.

## Conflict of Interest

The authors declare that the research was conducted in the absence of any commercial or financial relationships that could be construed as a potential conflict of interest.

## Publisher’s Note

All claims expressed in this article are solely those of the authors and do not necessarily represent those of their affiliated organizations, or those of the publisher, the editors and the reviewers. Any produt that may be evaluated in this article, or claim that may be made by its manufacturer, is not guaranteed or endorsed by the publisher.
